# Gestational diabetes impacts fetal precursor cell responses with potential consequences for offspring

**DOI:** 10.1002/sctm.19-0242

**Published:** 2019-12-27

**Authors:** Francisco Algaba‐Chueca, Elsa Maymó‐Masip, Miriam Ejarque, Mónica Ballesteros, Gemma Llauradó, Carlos López, Albert Guarque, Carolina Serena, Laia Martínez‐Guasch, Cristina Gutiérrez, Ramón Bosch, Joan Vendrell, Ana Megía, Sonia Fernández‐Veledo

**Affiliations:** ^1^ Servei d'Endocrinologia i Nutrició i Unitat de Recerca Hospital Universitari de Tarragona Joan XXIII Tarragona Spain; ^2^ Institut d'Investigació Sanitària Pere Virgili (IISPV) Tarragona Spain; ^3^ CIBER de Diabetes y Enfermedades Metabólicas Asociadas (CIBERDEM)‐Instituto de Salud Carlos III Madrid Spain; ^4^ Servei de Ginecologia i Obstetricia Hospital Universitari de Tarragona Joan XXIII Tarragona Spain; ^5^ Department of Endocrinology and Nutrition Hospital del Mar, Institut Hospital del Mar d'Investigacions Mèdiques (IMIM) Barcelona Spain; ^6^ Department of Pathology Plataforma de Estudios Histológicos, Citológicos y de Digitalización, Hospital de Tortosa Verge de la Cinta Tortosa Spain; ^7^ Departament de Medicina i Cirurgia Universitat Rovira i Virgili Reus Spain

**Keywords:** fetal precursors, gestational diabetes, offspring, placenta, programming, stem cells

## Abstract

Fetal programming has been proposed as a key mechanism underlying the association between intrauterine exposure to maternal diabetes and negative health outcomes in offspring. To determine whether gestational diabetes mellitus (GDM) might leave an imprint in fetal precursors of the amniotic membrane and whether it might be related to adverse outcomes in offspring, a prospective case‐control study was conducted, in which amniotic mesenchymal stem cells (AMSCs) and resident macrophages were isolated from pregnant patients, with either GDM or normal glucose tolerance, scheduled for cesarean section. After characterization, functional characteristics of AMSCs were analyzed and correlated with anthropometrical and clinical variables from both mother and offspring. GDM‐derived AMSCs displayed an impaired proliferation and osteogenic potential when compared with control cells, accompanied by superior invasive and chemotactic capacity. The expression of genes involved in the inflammatory response (*TNFα*, *MCP‐1*, *CD40*, and *CTSS*) was upregulated in GDM‐derived AMSCs, whereas anti‐inflammatory *IL‐33* was downregulated. Macrophages isolated from the amniotic membrane of GDM mothers consistently showed higher expression of *MCP‐1* as well. In vitro studies in which AMSCs from healthy control women were exposed to hyperglycemia, hyperinsulinemia, and palmitic acid confirmed these results. Finally, genes involved in the inflammatory response were associated with maternal insulin sensitivity and prepregnancy body mass index, as well as with fetal metabolic parameters. These results suggest that the GDM environment could program stem cells and subsequently favor metabolic dysfunction later in life. Fetal adaptive programming in the setting of GDM might have a direct negative impact on insulin resistance of offspring.

## Graphical abstract

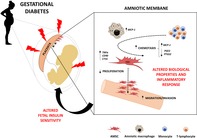

**Significance statement**

Signatures of metabolic deregulation seem to remain in cells early in development. Given the location on the inner side of the placenta, amniotic membrane stem cells might be a good indicator of how the intrauterine environment impacts the fetus. To the best of authors' knowledge, this study showed for the first time how gestational diabetes disturbs both the phenotype and the functional characteristics of amniotic mesenchymal stem cells, and these alterations are related to maternal and fetal metabolic status, suggesting that fetal adaptive programming in the setting of gestational diabetes might have a direct impact on offspring.

## 1 INTRODUCTION

Gestational diabetes mellitus (GDM) is associated both with short‐term adverse obstetric and perinatal complications and with long‐term metabolic health consequences for offspring.^1^ In this context, fetal programming has been proposed as a key mechanism underlying the link between the intrauterine exposure to maternal diabetes and an increased risk for metabolic dysfunction in adulthood, leading to type 2 diabetes, obesity, and cardiovascular disease.^2^ However, the ultimate mechanisms involved are not well known.

As a natural interface between mother and fetus, the functional integrity of the placenta is crucial to support fetal growth and development as well as to adapt to the maternal nutritional and metabolic status.^3^ Thus, beyond its recognized endocrine, immunological, and vascular functions, the placenta is a highly structured organ with multiple cell types dedicated to nutrient transport and energy metabolism.^4^ Although the extent to which maternal glycemic control contributes to placenta dysfunction remains a matter of debate,^5^ an enlarged size, altered vascular permeability, and the presence of low‐grade inflammation seem to be typical features of GDM.^4^ In this aspect, the potential of these disturbances to imprint fetal precursor cells found in the placenta and/or umbilical cord is emerging as a novel pathway playing a key role in the etiology of metabolic diseases in offspring of mothers with GDM. Indeed, recent studies have revealed the significant impact of GDM on human umbilical cord‐derived stromal cells in terms of proliferation,^6^ mitochondrial dysfunction,^7^ and angiogenesis.^8^

The amniotic membrane is the innermost layer of the placenta and possesses anti‐inflammatory and antibacterial properties that contribute to materno‐fetal tolerance. It also exerts many metabolic functions, such as the transport of water and soluble molecules and the production of bioactive factors.^9^ Our study sought to investigate whether GDM might leave an imprint in fetal precursors found in the amniotic membrane and, if so, whether it might be related to adverse outcomes in offspring. More specifically, we focused on amniotic mesenchymal stem/stromal cells (AMSCs) which are mainly characterized by their low immunogenicity, immunomodulatory properties and mesodermal multilineage differentiation capacity in vitro.^10^ We report evidence that maternal metabolic derangements during gestation disturb the biological properties of AMSCs. Of note, we found an association between the biological features of these fetal precursor cells and the maternal and fetal clinical and metabolic parameters, supporting the notion that fetal adaptive programming in the setting of GDM might have a direct impact on offspring.

## 2 MATERIAL AND METHODS

### 2.1 Study subjects

Eighteen pregnant women with a singleton pregnancy (9 with GDM and 9 with normal glucose tolerance acting as controls) scheduled for cesarean delivery were included in this study, which was performed at the Hospital Universitari de Tarragona Joan XXIII (HUJ23) according to the tenets of the Helsinki Declaration. The institutional review board (CEIm) approved the study protocol and all patients gave written informed consent before participating in the study. All mothers diagnosed with GDM were recruited at the Diabetes and Pregnancy Clinic of the HUJ23, while control subjects were recruited at the delivery suite. GDM was diagnosed according to the current criteria of the Spanish Diabetes in Pregnancy guidelines, which followed the National Data Group Criteria.^11^, ^12^ Four of the GDM women were treated only with diet whereas five were also treated with insulin. Timing of delivery was based primarily on obstetric indications. Gestational age was confirmed in all pregnant women by a routine ultrasonographic examination performed before 20 weeks of gestation. Exclusion criteria for all subjects were preexisting type 1 or type 2 diabetes, inflammatory or chronic diseases, current use of drugs known to affect carbohydrate metabolism, fetal anomalies identified at birth, smoking, or high blood pressure.

### 2.2 Clinical and demographic data

Upon inclusion, demographic and historical data were collected paying special attention to third trimester HbA1c in GDM women, 1‐hour 50‐g glucose challenge test (GCT), pregravid weight, gestational weight gain (GWG), and gestational age at delivery. Maternal anthropometric measurements of height (measured to the nearest 0.5 cm) and weight (measured to the nearest 0.1 kg) were obtained using a medical scale. Body mass index (BMI) was calculated using the formula BMI = weight (in kilograms)/height (in meters)^2^. GWG was calculated as (final weight)−(pregravid weight). Neonatal length, weight, and waist circumference were measured at birth and the waist circumference/length ratio was calculated. Suprailiac skinfold thickness was measured at least three times using a Holtain skinfold caliper (Chasmors Ltd, London, UK) to obtain a consistent and stable reading.

### 2.3 Sample collection and processing

Maternal blood samples were collected in the morning after an 8 hours fast and immediately before cesarean section, and umbilical vein cord blood was obtained at the time of delivery. Serum was immediately separated by centrifugation and stored at −80°C until analysis. Full‐term placentas (37–39 weeks gestation) were collected after delivery and immediately processed under sterile conditions. The amniotic membrane was mechanically peeled free from underlying chorion and washed with phosphate‐buffered saline (PBS) containing antibiotics.

### 2.4 Laboratory measurements

Glucose, cholesterol, and triglycerides levels were determined using ADVIA 1800 and 2400 (Siemens AG, Munich, Germany) autoanalyzers by standard enzymatic methods. Fasting plasma insulin was determined by immunoassay on the Centaur XP platform (Siemens AG). Homeostasis model assessment‐insulin resistance (HOMA‐IR) was determined according to the equation HOMA‐IR = fasting plasma glucose (mmol/L) × fasting plasma insulin (μU/mL)/22.5. HbA1c was estimated by high‐performance liquid chromatography‐based ion exchange chromatography (ADAMS‐A1c HA‐8160; Menarini Diagnostics, Florence, Italy).

### 2.5 Histological study of placenta

Tissue sections from the maternal (facing the decidua) and fetal (facing the amniotic cavity) sides of the placentas were collected, washed, fixed in a 4% paraformaldehyde, embebbed in paraffin, cut into slices using a rotator microtome and mounted onto glass microscope slides. Tissue sections were then dewaxed and stained with Masson's trichrome to highlight connective tissue and collagen fibers of the extracellular matrix. Slides were observed microscopically at ×2.5 magnification using a Leica DM LB2 bright‐field microscope (Leica Microsystems GmbH, Wetzlar, Germany). Ten different nonoverlapping fields randomly selected from each slide were visualized and captured at ×40 magnification using a Leica DFC320 Digital Camera (Leica Microsystems). Morphometric measurements were performed using Image‐Pro Plus 5.0 software (MediaCybernetics Inc., Silver Springs, Maryland) programmed for multistep algorithms: one for the detection of the total area of the villous/image (in pixels) and a second for the detection of the total trichrome‐positive green pixels/image (fibrotic area). The ratio between the fibrotic area and the total area of the villous was calculated for each image. Finally the average of the ratios of the 10 images was calculated for each case.

### 2.6 Isolation and culture of human amniotic mesenchymal stem cells

AMSCs were isolated as previously described.^13^, ^14^ Briefly, the amniotic membrane was digested in a rotary incubator at 37°C with a solution of 0.25% trypsin‐EDTA for 30 minutes and then with collagenase type IV solution (Gibco, Carlsbad, California) in complete medium comprising Dulbecco's modified Eagle's medium (DMEM)/F12, 10% fetal bovine serum (FBS) and 1% antibiotic/antimycotic solution, for 1.5 hours. After centrifugation and washing, cell pellet was resuspended in complete medium. Primary cultures of AMSCs at passage 0 were grown to 80%‐90% confluence and harvested with 0.25% trypsin‐EDTA. Non‐adherent cells were removed by rinsing twice with PBS. All of the experiments were performed at passages 3‐5 to ensure cell purity. For chemotaxis experiments and prostaglandin E2 experiments, 24‐hour culture media containing 0.2% BSA and without FBS were collected and stored at −80°C.

### 2.7 Isolation of amniotic membrane‐resident macrophages

Macrophages were purified by adherence to tissue culture plates following an established procedure that results in >90% purity.^15^ Briefly, the cell pellet from the digested membrane was resuspended in complete medium and allowed to attach for 10 minutes. The supernatant was then collected and seeded again in different flasks to obtain AMSCs. Culture plates were then washed twice with PBS. Cells that did not express the macrophage marker CD68 were discarded.

Since amniotic macrophages and AMSCs are isolated from the same primary sample, in order to assess their purity, the expression levels of typical markers of monocyte‐lineage and antigen presenting cells, such as *CD80*, *CD86*, *CD163*, *CD206*, and *CD209* were compared in amniotic macrophages and AMSCs.

### 2.8 Immunophenotyping

To assess mesenchymal lineage features and to determine whether they met the minimum criteria defined by the International Society of Cell Therapy,^16^ AMSCs (1 × 10^5^) were incubated with a panel of primary antibodies (BD Pharmingen, San Diego, California) and their surface markers expression was analyzed by flow cytometry (FacsAriaIII, BD Biosciences, San Jose, California). Data analysis was performed using the FACSDiva software (BD Biosciences).

### 2.9 Multilineage differentiation capacity

To determine the multilineage differentiation capacity of AMSCs, specific differentiation conditions were used to trigger cell differentiation into adipocytes, chondrocytes, and osteocytes as described.^17^, ^18^ Differentiated cells were stained with Oil Red O, Alcian Blue or Alizarin Red, respectively, and observed in a bright‐field microscope (ZEISS Axio Vert A1, Oberkochen, Germany). Quantification of the differentiation capacity was assessed by extracting the dyes from cell cultures (isopropanol for Oil Red O and cetylpyridinium chloride for Alizarin Red) and by measuring their absorbance by spectrophotometry at 540 nm.

### 2.10 Migration capacity

The migratory capacity of AMSCs was determined using a Transwell system (8‐μm polycarbonate membrane; Corning, New York, New York), as described.^19^ Briefly, lower chambers were filled with DMEM high glucose 0.2% BSA and cells (8 × 10^4^) suspended in the same medium were added to the upper chambers. Transwells were then incubated for 24 hours at 37°C. Cells that migrated to the lower chamber were then collected and counted using the BioRad TC 10 Automated Cell Counter (BioRad, Hercules, California).

### 2.11 Invasion capacity

The invasive capacity of AMSCs was determined as for the migration assay except that Transwell membranes were firstly coated with Matrigel (Corning) in DMEM high glucose 0.2% BSA for 2 hours at 37°C. Cells (8 × 10^4^) were added to the upper chambers and incubated for 24 hours at 37°C and those ones that invaded into the lower chamber were collected and counted as above.

### 2.12 Stimulation experiments

AMSCs from control pregnant women were cultured in 6‐well plates (5 × 10^4^) and allowed to attach for 24 hours. Cells were then stimulated for 24 hours with glucose, insulin and/or palmitic acid (PA) to a final concentration of 30 mM, 100 nM, and 0.4 mM, respectively. Absolute ethanol (EtOH) was used as vehicle to dissolve palmitic acid so a control of EtOH 0.4 mM was added in all the experiments. All conditions including PA were relativized to that control.

### 2.13 Chemotaxis capacity

The migratory response of human monocytes (THP‐1 cell line) and human T lymphocytes (Jurkat cell line) to the conditioned medium of AMSCs was determined as for the migration assay, except that a 5‐μm polycarbonate membrane was used. Lower chambers were filled with 24‐hour conditioned medium without FBS. In the stimulation experiments, 24‐hour conditioned medium was collected 24 hours after removing the stimuli. THP‐1 or Jurkat cells (1 × 10^5^) suspended in DMEM/F12, 0.1% BSA were then added to the upper chambers and incubated overnight at 37°C. Cells that migrated to the lower chambers were collected and counted as above.

### 2.14 MCP‐1 blockage experiments

MCP‐1 neutralization was performed as per the chemotaxis experiments with THP‐1 cells, except that the 24‐hour conditioned media without FBS were incubated with 20 μg/mL of an antibody against MCP‐1 (CCL2 [MCP‐1] Monoclonal Antibody 5D3‐F7, eBioscience) for 30 minutes at room temperature before being added to the lower chamber of the Transwell System. A negative epitope control (Mouse IgG1 kappa Isotype Control, eBioscience) was included in each experiment. THP‐1 cells (1 × 10^5^) suspended in DMEM with 0.2% BSA were then added to the upper chambers and incubated overnight at 37°C. Cells that migrated to the lower chambers were collected and counted as mentioned above.

### 2.15 Cell proliferation

Proliferation rate of AMSCs was determined by standard colorimetric 3‐(4,5‐dimethylthiazol‐2‐y1)‐2,5‐diphenyltetra‐zolium bromide (MTT) incorporation experiments. Cells (1.6 × 10^3^) were cultured in 96‐well plates and allowed to attach for 24 hours. A MTT assay at day 1 was performed to count the initial number of cells. After 5 days, a second MTT assay was performed (day 7) and the difference in absorbance between day 7 and day 1 was considered the proliferation rate. In the AMSCs stimulation studies, the proliferation rate was measured at 24 hours after the addition of the stimuli. Absorbance was measured by spectrophotometry at 540 nm in all cases.

### 2.16 Prostaglandin E_2_ determination

Prostaglandin E_2_ (PGE2) concentrations were measured, at 24 hours, in the conditioned medium of AMSCs by ELISA (R&D Systems) following the manufacturer's instructions.

### 2.17 Gene expression analysis

Total RNA was isolated from cells using the RNeasy Mini kit (Qiagen, Valencia, California) and its quality was assessed by the OD260/OD280 ratio. For gene expression analysis, RNA was transcribed into cDNA with random primers using the Reverse Transcription System (Applied Biosystems, Foster City, California). Quantitative gene expression was evaluated by real‐time polymerase chain reaction (RT‐PCR) on a 7900HT Fast Real‐Time PCR System using the following predesigned TaqMan primers (Applied Biosystems): *18S* (Hs03928985_g1), *ACACA* (Hs01046047_m1), *ALP* (Hs01029144_m1), *CCL3* (Hs00234142_m1), *CD40* (Hs01002915_g1), *CD74* (Hs00269961_m1), *CD80* (Hs01045161_m1), *CD86* (Hs01567026), *CD163* (Hs00174705), *CD209* (Hs01588349), *COL1a1* (Hs00164004_m1), *COL2a1* (Hs00264051_m1), *COMP* (Hs00164359_m1), *CTSB* (Hs00947439_m1), *CTSS* (Hs00175407_m1), *FABP4* (Hs01086177_m1), *IL‐1β* (Hs01555410_m1), *IL‐6* (Hs00174131_m1), *IL‐10* (Hs00961622_m1), *IL‐12b* (Hs01011518_m1), *IL‐33* (Hs04931857_m1), *LPL* (Hs00173425_m1), *MCP‐1* (Hs00234140_m1), *MRC‐1* (Hs00267207), *PPAR*γ (Hs01115513_m1), *PTGS2* (Hs00153133), *TGFβ1* (Hs00998133_m1), *TNFα* (Hs00174128_m1).

### 2.18 Statistical analysis

Data were analyzed with SPSS software version 21.0 (IBM, Armonk, New York). The one‐sample Kolmogorov‐Smirnov test was performed to verify normal distribution of the quantitative variables. Quantitative variables are expressed as mean ± SD or SEM as indicated and categorical variables are reported as number (percentages). Student's *t* test was used to compare the mean values of continuous variables. To analyze differences in nominal variables between groups we used the chi‐squared test. Pearson's correlation coefficient was used to analyze the univariate correlation between gene expression and clinical and metabolic parameters. A *P*‐value <.05 was considered statistically significant in all analyses.

## 3 RESULTS

### 3.1 Clinical and placental histological data of the population studied

AMSCs were isolated from the amniotic membrane of mothers with a healthy pregnancy (control group, n = 9) and with GDM (n = 9). Clinical, anthropometric, and biochemical data from mothers and offspring are presented in Table 1. In the GDM group, no differences in maternal HOMA‐IR and insulin concentrations were observed between diet and insulin treated women.

**Table 1:** Clinical characteristics of the subjects included in the study

	Whole group (18)	Control (9)	GDM (9)	*P*
Maternal age, years	35.06 ± 5.34	34.11 ± 6.49	36.00 ± 4.06	.470
Parity ≥1, n (%)	10 (55.6)	4 (44.4)	6 (66.7)	.603
Prepregnancy BMI, kg/m^2^	26.42 ± 4.43	24.85 ± 3.31	28.00 ± 5.03	.067
Gestational weight gain, kg	10.09 ± 5.10	11.83 ± 5.29	8.14 ± 4.37	.081
1‐h 50‐g glucose challenge, mg/dL	148.94 ± 37.35	120.44 ± 24.11	177.44 ± 23.57	<.001
Maternal glucose, mg/dL	79.23 ± 12.69	77.57 ± 7.68	81.17 ± 17.53	.632
HbA1c %	‐	‐	5.36 ± 0.25	
Maternal HOMA‐IR	2.75 ± 1.93	1.70 ± 3.98	3.98 ± 2.20	.052
Maternal insulin	14.20 ± 8.31	10.13 ± 4.23	18.27 ± 9.59	.061
Maternal total cholesterol, mg/dL	212.31 ± 38.21	224.71 ± 42.59	197.83 ± 29.35	.220
Maternal triglycerides, mg/dL	224.38 ± 82.76	244.00 ± 87.93	201.50 ± 77.41	.379
Week at delivery, weeks	38.17 ± 0.99	37.78 ± 0.83	38.56 ± 1.01	.097
Newborn weight, g	3,396 ± 454	3,385 ± 448	3,408 ± 487	.517
Placental weight, g	661.67 ± 130.45	625 ± 66	698 ± 170	.460
Suprailiac skinfold, mm	3.35 ± 0.49	3.29 ± 0.39	3.42 ± 0.60	.917
Cord blood glucose, mg/dL	68.06 ± 11.96	64.25 ± 12.07	71.87 ± 11.32	.213
Infant male sex, n (%)	9 (50%)	5 (55.6)	4 (44.4)	.637
Cord blood insulin	6.70 ± 4.61	6.04 ± 3.53	7.35 ± 5.63	.567
Cord blood total cholesterol, mg/dL	60.44 ± 8.97	56.00 ± 3.70	64.88 ± 10.66	.054
Cord blood triglycerides, mg/dL	29.56 ± 18.30	23.75 ± 4.95	33.38 ± 24.82	.215

**Figure 1:**
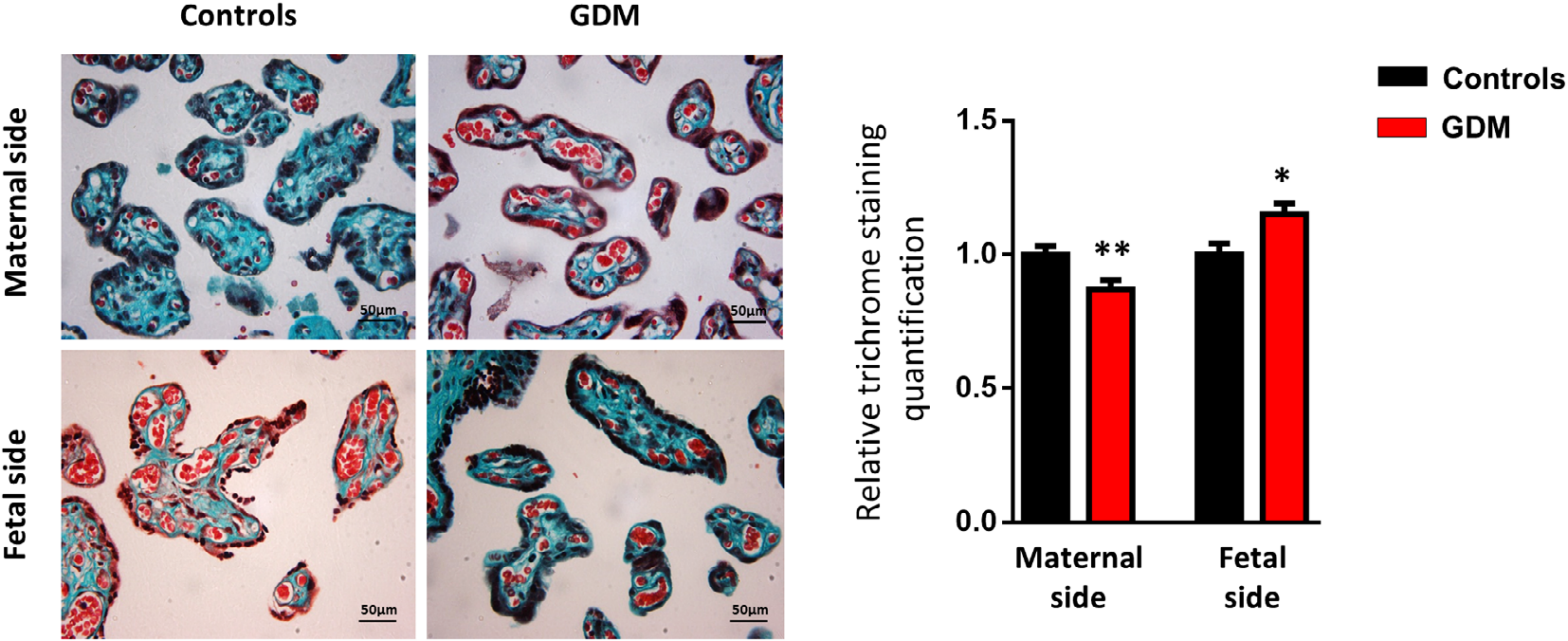
Histological features of the placental tissue from maternal and fetal sides obtained from gestational diabetes mellitus (GDM) and control women. Representative photomicrographs of terminal and intermediate villi in placental sections from pregnant control and GDM women, stained with Masson's trichrome to highlight connective tissue and collagen fibers (in green). Quantification of Masson's trichrome staining in villous stroma of placental sections is shown as percentage mean area ± SD (n = 6‐8 per group). Results are shown as mean ± SEM from independent donor experiments performed in duplicate. *P < .05 vs controls, **P < .01 vs controls

Placental abnormalities associated with GDM have been inconsistently reported in the literature, perhaps reflecting glycemic control, and prenatal care quality. First, we assessed macroscopic and histopathologic features of the collected placentas. The macroscopic study of the placentas failed to find difference between GDM and control groups. No differences were observed in placental weight and area (Table 1), and the umbilical cord was marginally inserted in most of the placentas studied (7/9 of the control group and in 5/9 of the GDM group). However, the histological study of placental tissue showed differences in the distribution of collagen deposition in villous stroma between groups. Staining quantification indicated lower collagen deposition on the maternal side of placentas obtained from GDM women (*P* = .0173) whereas it was higher on the fetal side compared the controls (*P* = .0067) (Figure 1). These results indicate that a diabetic environment modifies the collagen deposition within the placenta, highlighting the presence of fibrosis on the placenta's fetal side, which is characterized by an excessive connective tissue accumulation in response to tissue injury and inflammation.

### 3.2 Isolation and characterization of amniotic mesenchymal stem cells from GDM and control women

**Figure 2:**
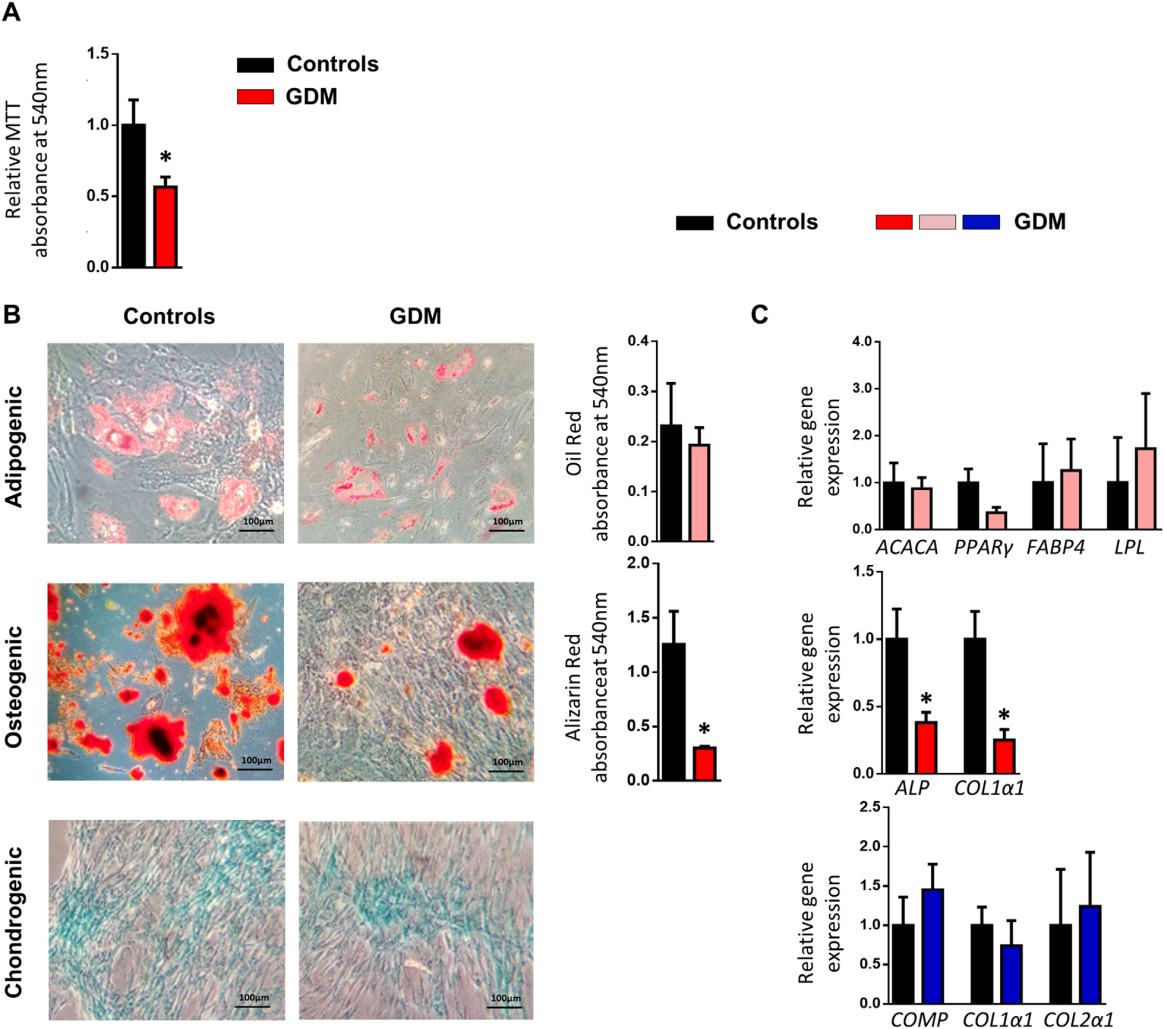
Gestational diabetes mellitus (GDM) affects the plasticity of fetal precursor cells from the amniotic membrane. A, MTT incorporation in proliferating amniotic mesenchymal stem cells (AMSCs) isolated from pregnant control and GDM women (n = 7‐9 per group). B, Representative photomicrographs of AMSCs isolated from pregnant control and GDM women (n = 4 per group), differentiated into adipocytes, osteocytes, and chondrocytes and stained with Oil Red, Alizarin Red, and Alcian Blue, respectively (magnification ×200). Quantification of the differentiation capacity was assessed by extracting the staining dyes and measuring the absorbance by spectrophotometry at 540 nm. C, Gene expression of adipogenic (*ACACA*, *PPARy*, *FABP4*, *LPL*), osteogenic (*ALP*, *COL1α1*), and chondrogenic (*COMP*, *COL1α1*, *COL2α1*) markers in AMSCs isolated from pregnant control and GDM women (n = 4 per group). In all cases, values of differentiated cells were normalized to their undifferentiated counterparts. Results are shown as mean ± SEM from independent donors experiments performed in duplicate. *P < .05 vs controls

Flow cytometry analysis of cell marker expression was consistent with the minimum criteria defined for AMSCs. Accordingly, cells were positive for the surface markers CD90, CD73, and CD105 and negative for CD45, CD34, CD31, CD14, and the human leukocyte antigen complex DR (HLA‐DR) (Table 2). No significant differences were detected between groups. Likewise, the amniotic membrane‐cell number ratio (number of cells obtained per gram of tissue) was similar between control and GDM women (data not shown). By contrast, cell proliferation was significantly lower in AMSCs isolated from GDM mothers as measured by MTT incorporation assays (Figure 2A). Both groups of AMSCs demonstrated similar adipogenic and chondrogenic differentiation capacities, typical of mesodermal cells. However, AMSCs from GDM mothers showed an impairment in the osteogenic lineage differentiation as revealed by a diminished Alizarin Red staining quantification and gene expression of typical osteogenic markers including alkaline phosphatase (*ALP*) and collagen type I alpha 1 chain (*COL1a1*) (Figure 2B,C).

**Table 2:** Immunophenotypic profile of AMSCs from pregnant control and gestational diabetes mellitus (GDM) women

Marker	Control (%)	GDM (%)
CD90	97.88 ± 1.35	96.02 ± 3.28
CD73	94.18 ± 5.36	93.28 ± 4.41
CD105	91.78 ± 5.40	88.24 ± 7.01
CD45	0.02 ± 0.045	0.025 ± 0.05
CD34	0 ± 0	0.24 ± 0.33
CD31	0.366 ± 0.45	0.26 ± 0.207
CD14	0.34 ± 0.47	0.34 ± 0.53

### 3.3 GDM displays an immune response in AMSCs

**Figure 3:**
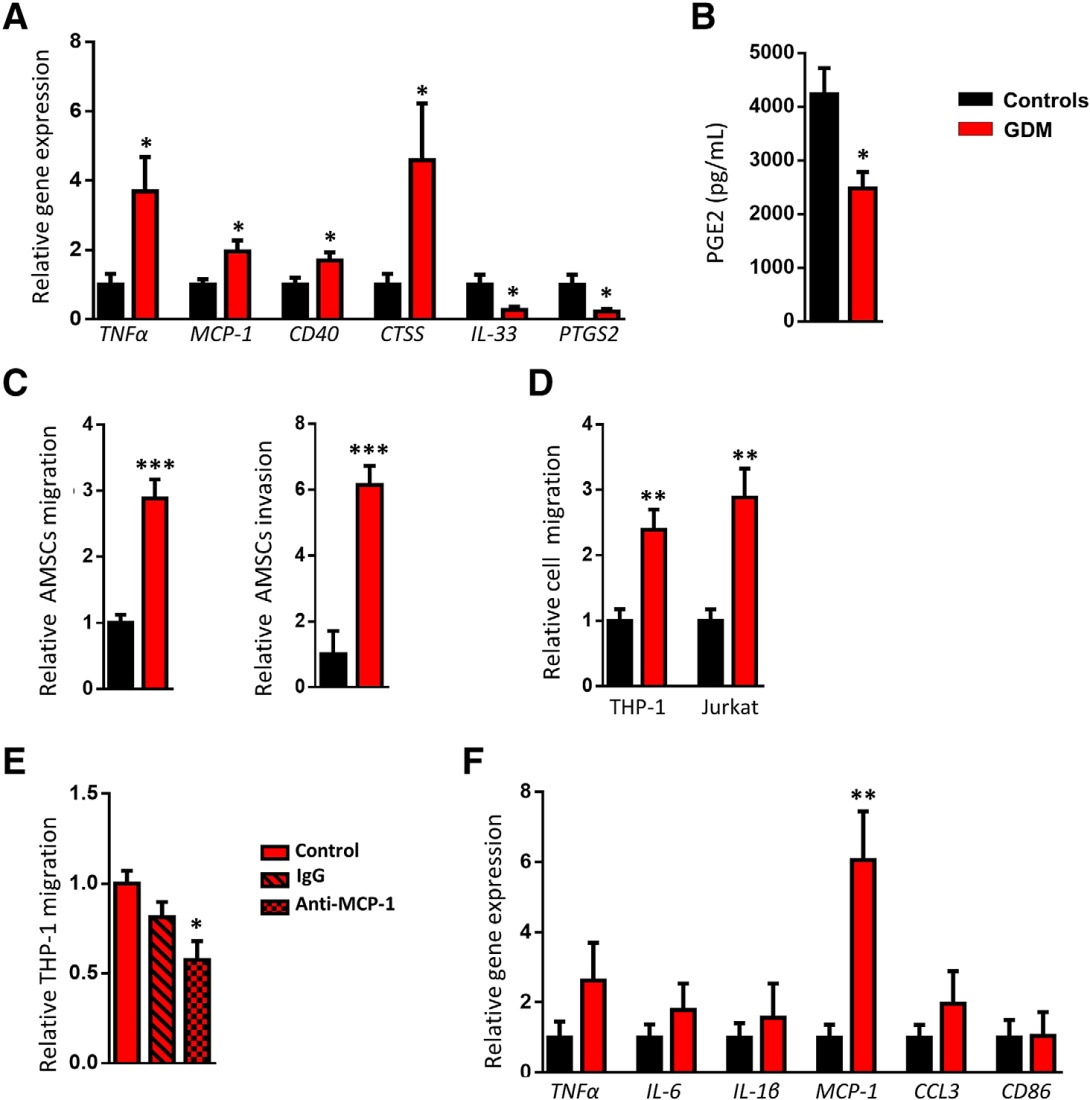
Disturbances in amniotic mesenchymal stem cells (AMSCs) and amniotic membrane‐resident macrophages from placentas obtained from gestational diabetes mellitus (GDM) women. A, Gene expression analysis of the inflammatory markers *TNFα*, *MCP‐1*, *CD40*, *CTSS*, *IL‐33*, and *PTGS2* in AMSCs isolated from pregnant control and GDM women (n = 8 per group). B, Prostaglandin E_2_ (PGE2) levels in the conditioned medium of AMSCs from pregnant control and GDM women (n = 6‐7 per group). C, Migratory and invasive capacities of AMSCs isolated from pregnant control and GDM women assessed in Transwell assays (n = 7‐9 per group). D, Migration of THP‐1 and Jurkat cells to AMSC‐conditioned medium assessed in Transwell assays (n = 5‐7 per group). E, Migration of THP‐1 cells to GDM‐AMSC‐conditioned medium after incubation with anti‐MCP‐1 antibody. F, Gene expression analysis of pro‐inflammatory and chemotactic markers (*TNFα*, *IL‐6*, *IL‐1β*, *MCP‐1*, *CCL3*, and *CD86*) in amniotic membrane‐resident macrophages from pregnant control and GDM women (n = 6‐8 per group). Results are shown as mean ± SEM from independent donor experiments performed in duplicate. *P < .05 vs controls, **P < .01 vs control, ***P < .0001 vs controls

A gene expression signature of placental inflammation in pregnancies complicated by GDM has been previously reported. However, the cellular events underlying this process remain unknown or, at least, unclear. The GDM‐AMSCs inflammatory expression profile presented an increase in the expression of genes encoding the pro‐inflammatory cytokine tumor necrosis factor alpha (*TNFα*) and the chemokine monocyte chemoattractant protein 1 (*MCP‐1*), as well as in genes involved in the inflammatory response such as *CD40* and cathepsin S (*CTSS*) compared to controls (Figure 3A). No differences were observed in the expression of interleukin 10 (*IL‐10*), transforming growth factor beta 1 (*TGFβ1*), *CD74*, *CD80*, or cathepsin B (*CTSB*) (data not shown). By contrast, GDM‐AMSC showed a significant reduction in the expression of *IL‐33*, a cytokine with anti‐inflammatory properties, and also of prostaglandin‐endoperoxide synthase 2 (*PTGS2*), a key enzyme in prostaglandin biosynthesis (Figure 3A). Accordingly, prostaglandin E2 (PGE2) levels were lower in the conditioned medium from GDM‐AMSCs compared to those from control cells (4237.28 ± 25.37 pg/mL vs 2476.62 ± 30.36 pg/mL, *P* = .0109) (Figure 3B). At the same time, these findings are accompanied by an increase in GDM‐AMSCs migratory and invasive capacities as compared to control cells (Figure 3C).

MCP‐1 is a potent chemoattractant and PGE2 has been involved in the anti‐inflammatory and immunosuppressive capacities of the AMSCs. According to these findings, the migration of monocytes and T‐lymphocytes was significantly higher when exposed to the conditioned medium from GDM‐AMSCs compared to those from control cells (Figure 3D) and this increased chemotactic capacity was significantly reversed when we treated the conditioned medium of GDM‐AMSCs with an anti‐MCP‐1 specific antibody (Figure 3E). In addition, amniotic membrane‐resident macrophages isolated from GDM mothers showed a higher expression of *MCP‐1*, a gene associated with a pro‐inflammatory (M1) phenotype (Figure 3F), whereas a similar but not significant trend was observed in the expression of pro‐repair (M2) markers such as *MRC‐1* (*CD206*), *CD163*, *CD209*, *peroxisome proliferator‐activated receptor gamma* (*PPARy*), and *IL‐10* between groups (Supplementary Figure S1). Collectively, these results indicate that GDM alters the inflammatory expression profile of cells with immunological function within the amniotic membrane, and specifically modifies the immunomodulatory properties of AMSCs.

### 3.4 In vitro stimulation with GDM‐like insults alters the inflammatory profile of control AMSCs

**Figure 4:**
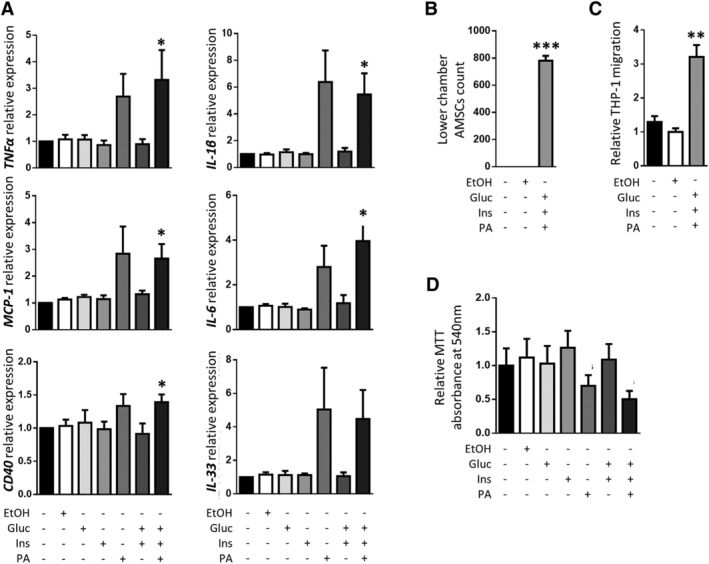
Phenotypic changes induced by a gestational diabetes mellitus (GDM)‐like environment rich in glucose, insulin, and palmitic acid on amniotic mesenchymal stem cells (AMSCs) obtained from control pregnant women. A, Gene expression analysis of the inflammatory markers *TNFα*, *MCP‐1*, *CD40*, *IL‐1β*, *IL‐6*, and *IL‐33* in AMSCs isolated from control pregnant women stimulated with glucose (Gluc), insulin (Ins), and/or palmitic acid (PA; n = 4 per group). B, Migration capability of control AMSCs stimulated with glucose, insulin, and/or PA assessed by Transwell assays (n = 3 per group). C, Migration of THP‐1 cells to the conditioned medium of control AMSCs stimulated with glucose, insulin, and/or PA assessed in Transwell assays (n = 4 per group). D, MTT incorporation in proliferating control AMSCs stimulated with glucose, insulin, and/or PA (n = 4 per group). *P < .05 vs control, **P < .01 vs controls, ***P < .0001 vs controls

Next, we assessed whether the exposure to hyperglycemia, hyperinsulinemia, and dyslipidemia, which are typical features of the GDM metabolic environment, modified the inflammatory profile of AMSCs. We stimulated in vitro AMSCs from four control women with high levels of glucose, insulin, or palmitic acid separately and also with different combination of them for 24 hours. No significant changes in the AMSCs inflammatory gene expression profile were observed when each stimuli was applied separately nor combining glucose and insulin, but nevertheless there was a significant increase in the expression of genes involved in the inflammatory response such as *TNFα*, *MCP‐1*, *CD40*, *IL‐1β*, and *IL‐6* when AMSCs were stimulated with the three stimuli together (Figure 4A). Moreover, AMSCs exposed to the three insults showed a significantly higher migratory capacity and an increased chemotactic activity over monocytes compared with the unstimulated ones (Figure 4B,C). Finally, in order to make sure that these results are specifically caused by the sum of the three stimuli tested and not by cell damage or death, we performed a MTT proliferation assay and found no significant differences in the proliferative capacity of the differentially stimulated AMSCs (Figure 4D). These data confirm that GDM adverse nutritional and metabolic environment alter the inflammatory profile of the amniotic membrane‐derived fetal precursors.

### 3.5 Biological properties of fetal precursor cells are related to maternal and infant clinical and metabolic parameters

Finally, we explored the potential relationship between maternal clinical and analytical parameters and AMSCs phenotype, and also whether their functional characteristics were associated with offspring's anthropometric and metabolic parameters. *MCP‐1* expression levels were correlated with maternal biomarkers associated with adverse perinatal and long‐term outcomes such as prepregnancy BMI and HOMA‐IR, as well as with cord blood concentrations of triglycerides and insulin. A positive association was also observed between *CD40* and *CTSS* with GCT and prepregnancy BMI (Table 3).

**Table 3:** Relationship observed between maternal and offspring clinical and metabolic parameters with amniotic mesenchymal stem cells (AMSCs) phenotype

	TNFα	MCP‐1	CD40	CTSS	IL‐33
Prepregnancy BMI	0.057 (0.821)	0.525 (0.025)	0.528 (0.024)	0.504 (0.033)	−0.195 (0.438)
1‐h 50 g challenge test	0.357 (0.159)	0.676 (0.003)	0.551 (0.022)	0.493 (0.044)	−0.232 (0.486)
Maternal glucose	−0.123 (0.688)	0.044 (0.888)	0.023 (0.940)	−0.102 (0.740)	−0.081 (0.749)
Maternal insulin	0.023 (0.941)	0.626 (0.022)	0.465 (0.108)	0.397 (0.179)	−0.003 (0.992)
Maternal HOMA‐IR	−0.005 (0.988)	0.648 (0.017)	0.479 (0.098)	0.353 (0.237)	−0.007 (0.979)
Maternal Triglycerides	−0.076 (0.805)	−0.344 (0.250)	−0.300 (0.320)	−0.125 (0.685)	−0.005 (0.986)
Suprailiac skinfold	−0.168 (0.520)	0.074 (0.779)	−0.041 (0.876)	−0.064 (0.806)	0.050 (0.842)
Cord blood glucose	0.194 (0.471)	0.309 (0.244)	0.335 (0.205)	0.268 (0.316)	−0.506 (0.032)
Cord blood insulin	−0.465 (0.070)	0.517 (0.040)	0.148 (0.584)	0.158 (0.559)	0.022 (0.931)
Cord blood triglycerides	0.234 (0.382)	0.535 (0.033)	0.496 (0.051)	−0.016 (0.952)	0.032 (0.901)

Next, we analyzed the groups separately and regarding the control group, all associations observed disappear except for *CD40* and cord blood triglycerides (*r*: 0.775; *P* = .014). On the other hand, in the GDM group, the correlations of *MCP‐1* with cord blood insulin (*r*: 0.745; *P* = .021) and GCT (*r*: 0.753; *P* = .019), became stronger, whereas its association with maternal HOMA‐IR (*r*: 0.638; *P* = .064) and prepregnancy BMI (*r*: 0.644; *P* = .061) followed a trend. The associations between *CD40* with maternal prepregnancy BMI (*r*: 0.683; *P* = .042) and GCT (*r*: 0.800; *P* = .010) remained significant. Overall, our data indicate that maternal metabolic phenotype during pregnancy could determine the biological characteristics of fetal precursors, which may be linked to metabolic characteristics in the offspring. These results become stronger in the context of GDM.

## 4 DISCUSSION

It has been proposed that signatures of metabolic deregulation remain in cells early in development.^20^ Given its location on the inner side of the placenta, the amniotic membrane is in contact with the amniotic fluid and the fetus and, as such, its stem cell component might be a good indicator of how the intrauterine environment impacts the fetus. Our results suggest that GDM modifies the plasticity of fetal precursor cells in the amniotic membrane. We show that GDM results in a deregulation of genes involved in inflammation in AMSCs, which have been associated with the development of insulin resistance, type 2 diabetes, obesity, and atherosclerosis, as well as with pro‐inflammatory changes of amniotic membrane‐resident macrophages. These data are supported by in vitro studies reproducing a GDM environment, in which the combination of hyperglycemia, hyperinsulinemia, and palmitic acid induced a similar phenotype in AMSCs obtained from control donors.

The novelty and the consistency of the clinical and experimental results are some of the strengths of our study. We have been able to reproduce our findings in vitro. AMSCs obtained from control women displayed an inflammatory gene expression profile similar to the pattern observed in GDM women when exposed to a GDM‐like environment. Furthermore, their migration and chemotactic capacities were affected. It is important to note that all placentas were obtained from a well‐characterized cohort of women scheduled for cesarean delivery in order to avoid any uncontrolled inflammatory stimuli linked to labor. Amniotic membrane samples were collected from the placental disk, halfway between the cord insertion point and the lateral edge, avoiding interregional differences. The sample size was similar to or even larger than other studies analyzing the plasticity of placental or umbilical cord mesenchymal stem cells.^6^, ^7^, ^8^, ^21^ However, it could be considered a limitation in the investigation of potential associations with newborn anthropometric variables, particularly because fetal growth is influenced not only by maternal metabolic and nutritional factors but also by the genetic potential and placental capacity to transfer nutrients. Nevertheless, *MCP‐1* is associated with maternal anthropometry and with maternal and fetal metabolic parameters.

Although usually discarded following birth, placental membranes are an easily accessible and nontraumatic source of mesenchymal stem cells (MSCs),^22^ and hence placental MSCs (pMSCs) are considered as a promising tool for cell‐based therapy.^9^ They can also shed light on how some diseases could modify intrauterine gene expression profiles. Data on the effect of GDM on pMSCs plasticity are scarce and somewhat discordant. The majority of studies have focused on chorionic^21^ and umbilical cord‐derived^6^, ^7^, ^8^ stem cells, and whereas a decreased clonogenic potential seems to be well establish, their differentiation ability is poorly defined.^6^, ^7^, ^8^, ^21^ Kim et al observed impaired differentiation in umbilical cord MSCs obtained from GDM women as compared with controls, in contrast to other reports.^8^, ^21^ We show that AMSCs obtained from GDM women retain their multipotent characteristics, albeit with an impaired osteogenic differentiation capability. According with our findings, Chen et al reported that umbilical cord MSCs isolated from obese women exhibited a poor cell differentiation potential to osteoblasts,^23^ and this was accompanied by a superior differentiation to adipocytes, which may favor adipogenesis. Therefore, our results support the hypothesis of maternal programming in GDM and identify AMSCs as new players in such a mechanism.

Previous reports showed that the conditioned medium from non‐GDM AMSCs induced macrophages to become M2‐like (anti‐inflammatory or pro‐repair) macrophages,^24^ and that macrophages of the placental villous stroma (Hofbauer cells) maintained an M2‐like phenotype in GDM,^25^ which is consistent with the role of placental tissue in inducing fetal‐maternal tolerance and for protection of fetus from low‐grade inflammatory environment. Our study found that macrophages obtained from the amniotic membrane of GDM mothers presented a higher expression of *MCP‐1* (and a notably trend in other pro‐inflammatory genes) than those obtained from controls, suggesting they have a more pro‐inflammatory profile predisposition, even in GDM women with a good metabolic control according to their third trimester HbA1c concentrations. Consistent with previous findings, the levels of PGE2, a potent immunomodulatory molecule secreted by mesenchymal stem cells deeply implied in their anti‐inflammatory and immunosuppressive capacities,^26^, ^27^, ^28^, ^29^ are lower in the conditioned media from GDM‐derived AMSCs. Likewise, these data are supported by a decreased expression of *PTGS2* in AMSCs from GDM women, a key enzyme involved in the PGE2 synthesis pathway.

A pro‐inflammatory state is known to be associated with the development of insulin resistance, obesity, type 2 diabetes, and atherosclerosis.^30^ *TNFα* expression is increased in placental tissue obtained from pregnancies complicated by GDM^31^ and it is also released by the placenta under hyperglycemic conditions.^32^ MCP‐1 is a key element involved in the modulation and recruitment of macrophages, participates in the induction of fat tissue inflammation in type 2 diabetes and obesity^33^ but also seems to have a pivotal role in adipogenesis.^34^ We have observed an increased expression of *MCP‐1* in AMSCs isolated from diabetic mothers along with an increased chemotactic capacity over monocytes and T‐lymphocytes through their conditioned medium. These phenomena may participate in the onset of placental inflammation that accompanies GDM. In addition to *TNFα* and *MCP‐1*, AMSCs isolated from GDM women showed an upregulation of genes involved in the inflammatory and immune response such as *CD40* and *CTSS*. Cathepsin is a cysteine protease implicated in the regulation of inflammatory activity and it has been postulated as a potential biomarker of the development of insulin resistance and type 2 diabetes.^35^ In agreement with epidemiological data, we observed an upregulation of *CTSS*, *TNFα*, and *MCP‐1* in AMSCs isolated from GDM mothers, suggesting intrauterine programming/imprinting.

Less consistent is the behavior of IL‐33, an interleukin involved in the maintenance of tissue homeostasis, resolution of inflammation, and repair of tissue damage.^36^ IL‐33 is considered to have pro‐ and anti‐inflammatory actions and it is released after cell injury, acting as an alarm signal that alerts immune cells about tissue damage.^37^, ^38^ Our results showed a decreased *IL‐33* expression in GDM‐derived AMSCs whereas in vitro control AMSCs exposed to a GDM‐like milieu showed an upward trend. Dalmas et al^39^ observed that IL‐33 was produced by islet mesenchymal cells in a diabetic milieu rich in glucose and palmitate but described a defective action in chronic obesity. Moreover, in some clinical studies IL‐33 concentrations showed different behavior in health and disease,^38^, ^39^ suggesting a downregulation in some chronic inflammatory diseases such as type 2 diabetes and obesity, limiting inflammatory damage.

Our in vitro results confirm that AMSCs exposed to high concentrations of insulin, glucose and palmitic acid, simulating the hyperinsulinemia, hyperglycemia, and dyslipidemia observed in metabolic syndrome^40^ but also in pregnancies complicated by diabetes,^41^ reproduced the inflammatory expression profile, the migration capacity, and the chemotactic activity observed in GDM‐derived AMSCs. Although we did not observe changes in the gene expression profile of control AMSCSs when exposed to insulin or glucose separately, a trend was seen when they were treated with palmitic acid. These results became evident when we treated them with a combination of the three stimuli, suggesting that saturated fatty acids were the main drivers of the metabolic activation but this effect was only significant in the context of hyperglycemia and hyperinsulinemia.

According to the fetal programming hypothesis,^42^ a disturbed metabolic environment could permanently affect the health of offspring exposed and predispose them to obesity and/or type 2 diabetes. GDM has been associated with an increased risk of obesity, type 2 diabetes, and cardiovascular disease in offspring, but the underlying pathogenic mechanisms are unclear. Despite GDM's association with macrosomia and excessive fat accretion, we found similar birth weight and adiposity in GDM offspring compared with control offspring. *MCP‐1* gene expression was positively correlated with both cord blood insulin and triglyceride concentrations, but not with birth weight or newborn adiposity. The lack of association between gene expression profiling and neonatal anthropometric parameters could be due, in part, to the sample size, the effect of nutritional therapy which normalizes fetal growth,^43^ and/or other factors besides hyperglycemia.^44^ On the other hand, we reported that maternal BMI and insulin resistance are related to gene expression of chemokines implicated in the modulation of migration and inflammation, but also involved in adipose tissue dysfunction and cardiovascular diseases. The upregulation of *MCP‐1* and *CD40* by the adverse maternal environment seems to be specific to pregnancies complicated by GDM and suggests that maternal nutritional and metabolic status could induce changes in the placental gene expression profile even when they are adequately controlled. It suggests that normalizing birth weight and metabolic control is unable to revert the placental insult. Based on this evidence, we speculate that GDM environment could program stem cells and subsequently favor metabolic dysfunction later in life.

## 5 CONCLUSION

For the first time, we demonstrate that maternal metabolic derangements in pregnancies complicated by GDM disturb both the phenotype and biological features of AMSC which are ultimately related to maternal and fetal nutritional and metabolic status, supporting the notion that fetal adaptive programming in the setting of GDM might have a direct impact on offspring. Our results also suggest that AMSCs might be a powerful tool for the indirect study of fetal cells in the context of hyperglycemia and insulin resistance, opening the possibility of new predictor or diagnostic approaches.

## CONFLICT OF INTEREST

The authors indicated no potential conflicts of interest.

## AUTHOR CONTRIBUTIONS

F.A.‐C.: conception and design, provision of study material or patients, collection and/or assembly of data, data analysis and interpretation, manuscript writing, final approval of manuscript; E.M.‐M.: conception and design, provision of study material or patients, collection and/or assembly of data, data analysis and interpretation, final approval of manuscript; M.E., R.B.: collection and/or assembly of data, data analysis and interpretation; M.B.: conception and design, provision of study material or patients, collection and/or assembly of data, final approval of manuscript, administrative support; G.L.: data analysis and interpretation; C.L., C.G.: collection and/or assembly of data, administrative support; A.G.: provision of study material or patients, collection and/or assembly of data; C.S.: data analysis and interpretation, article revision and final approval of manuscript; L.M.‐G.: provision of study material or patients, collection and/or assembly of data, article revision and final approval of manuscript; J.V.: final approval of manuscript, data analysis and interpretation, final approval of manuscript, administrative support, financial support; A.M.: conception and design, provision of study material or patients, collection and/or assembly of data, data analysis and interpretation, manuscript writing, final approval of manuscript, administrative support, financial support; S.F.‐V.: conception and design, data analysis and interpretation, manuscript writing, final approval of manuscript, administrative support, financial support.

## Supporting information


**Supplementary Figure 1** Gene expression analysis of pro‐repair markers (*MRC‐1*, *CD163*, *CD209*, *PPARγ* and *IL‐10*) in amniotic membrane‐resident macrophages obtained from pregnant control and GDM women (n = 6–8 per group).

## Data Availability

The data sets generated during and/or analyzed during the current study are available from the corresponding author on reasonable request.
