# Supplementary figures and images for: Gestational diabetes impacts fetal precursor cell responses with potential consequences for offspring
Source: Stem Cells Transl Med. 2019 Dec 27;9(3):351–63. doi: 10.1002/sctm.19-0242 (PMC7031647; doi:10.1002/sctm.19-0242)

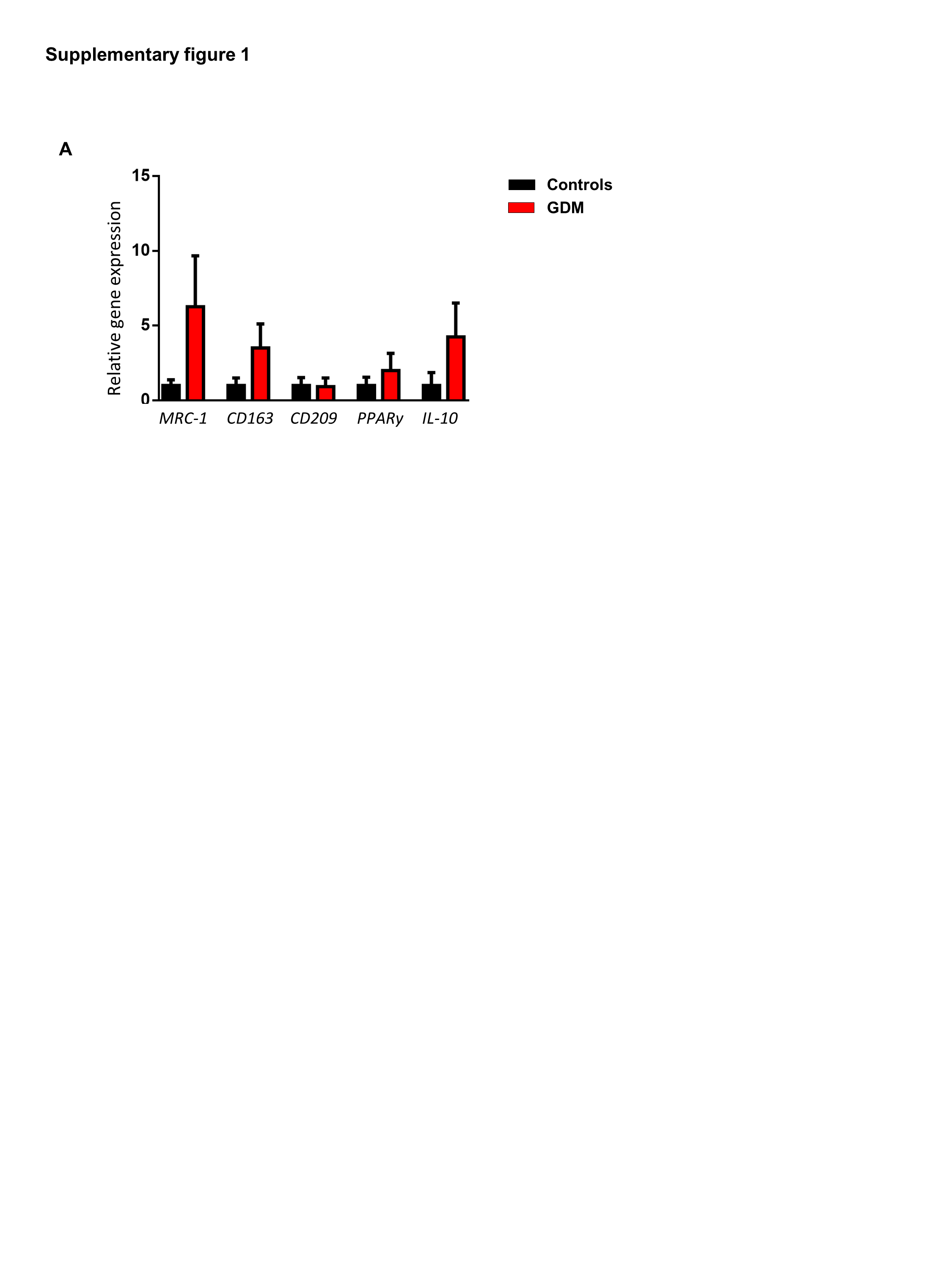

Supplement: Supplementary file 1 — Supplementary Figure 1 Gene expression analysis of pro‐repair markers (MRC‐1, CD163, CD209, PPARγ and IL‐10) in amniotic membrane‐resident macrophages obtained from pregnant control and GDM women (n = 6–8 per group). [file SCT3-9-351-s001.tif]
